# Undercorrection of hypernatremia is frequent and associated with mortality

**DOI:** 10.1186/1471-2369-15-37

**Published:** 2014-02-21

**Authors:** Stanislas Bataille, Camille Baralla, Dominique Torro, Christophe Buffat, Yvon Berland, Marc Alazia, Anderson Loundou, Pierre Michelet, Henri Vacher-Coponat

**Affiliations:** 1 Aix-Marseille University, APHM, Hôpital de la Conception, Centre de néphrologie et transplantation rénale, Marseille 13005, France; 2 Aix-Marseille University, APHM, Hôpital de la Conception, Service d’accueil des urgences, Marseille 13005, France; 3 Aix-Marseille University, APHM, Hôpital de la Conception, Laboratoire de Biochimie, Marseille 13005, France; 4 Aix-Marseille University, Laboratoire de Santé Publique EA3279, Marseille 13284, France

**Keywords:** Intracellular dehydration, Dysnatremia, Hypernatremia, Renal failure, Emergency, Mortality

## Abstract

**Background:**

About 1% of patients admitted to the Emergency Department (ED) have hypernatremia, a condition associated with a mortality rate of 20 to 60%. Management recommendations originate from intensive care unit studies, in which patients and medical diseases differ from those in ED.

**Methods:**

We retrospectively studied clinical characteristics, treatments, and outcomes of severely hypernatremic patients in the ED and risk factors associated with death occurrence during hospitalization.

**Results:**

During 2010, 85 cases of severe hypernatremia ≥150 mmol/l were admitted to ED. Hypernatremia occurred in frail patients: mean age 79.7 years, 55% institutionalized, 28% with dementia.

Twenty four percent of patients died during hospitalization. Male gender and low mean blood pressure (MBP) were independently associated with death, as well as slow natremia correction speed, but not the severity of hyperosmolarity at admission. Infusion solute was inappropriate for 45% of patients with MBP <70 mmHg who received hypotonic solutes and 22% of patients with MBP ≥70 mmHg who received isotonic solutes or were not perfused.

**Conclusions:**

This is the first study assessing outcome of hypernatremic patients in the ED according to the treatment provided. It appears that not only a too quick, but also a too slow correction speed is associated with an increased risk of death regardless of initial natremia. Medical management of hypernatremic patients must be improved regarding evaluation and treatment.

## Background

Hypernatremia is diagnosed in 1 to 2% of patients admitted to the Emergency Department (ED). Such patients often have various underlying pathologies implicated in hypernatremia occurrence [1]. In most cases, treatment of hypernatremia is started at ED, but there have been only two reports on the characteristics, symptoms, treatments, and outcomes of severely hypernatremic patients at ED [1,2].

The recommended hypernatremia correction speed, based on pediatric retrospective case-series [2], is 1 mmol/L/h when hypernatremia occurrence has been quick (a few hours) and 0.5 mmol/L/h when it has been long or is unknown [3-6]. A too fast correction can induce cerebral edema and irreversible neurologic sequels that are avoided with a slow correction [3,5,7]. Unless shock or hypotension are present, isotonic or hypertonic solutes are not recommended. Moreover, experts recommend first replacing fluid losses in dehydrated/hypovolemic patients by use of isotonic or slightly hypotonic fluids (e.g. lactated Ringer’s) [8].

To avoid over- or under-treatment, which both might cause neurological complications (cerebral edema, seizure, or coma), management of intravenous fluid must be rationalized. Mathematic calculation of water deficit based on sodium and water distribution in the intra- and extra-cellular spaces has been proposed. This requires two clinical data: natremia measurement and patient weight. For example, Adrogue and Madias’s formula serves to calculate natremia variation after perfusion of one liter of solute according to the type of solute, initial natremia, and total body water volume: *Attended natremia = ([Na] of solute – initial natremia)/(Total body water volume - 1)*[3,9,10]. Most formulas consider the human body as a closed system and do not integrate undergoing losses of water. Furthermore, total body water volume required for calculation is extrapolated from weight regardless of the percentage of body fat variability. Also, these formulas were found to be imprecise in individual patients with deviations > 10 mmol/L [11]. Their inappropriate use might lead to hypernatremia under-correction or worsening [12].

Mortality of hypernatremic patients reaches 20% to 60% [2,12-15] and varies according to comorbidities, associated illness, and whether hypernatremia is present at admission or acquired during hospitalization [2,16]. Hypernatremia at admission or acquired during the hospitalization is an independent mortality risk factor [2,15-17].

In this study, we report clinical characteristics, management, outcome and mortality risk factors of severely hypernatremic patients admitted to the ED.

## Methods

We retrospectively studied all patients admitted between January 2010 and January 2011 to the main adult ED of the center of Marseille, France. Each day an average of 150 patients are admitted. The medical team was constituted at daytime of 4 senior doctors and 5 residents and at night 3 senior doctors and 5 residents.

Patients were identified using the computerized registry of the biochemistry lab. All patients with natremia ≥150 mmol/L at admission were included in the study. Data collected from medical files were age, gender, place of origin and personal environment (home, institution or home medical care), cause of admission, blood pressure, presence of extracellular dehydration signs (decreased skin turgor, lower limb edema, hypotonic ocular globes), presence of neurologic symptoms (disorientation, agitation, somnolence, coma), medical history of dementia, usual treatments, sequential serum sodium measured during the hospitalization, creatinine at admission, ED main diagnosis, and patient outcome in ED and during the following hospitalization. We also collected composition and perfusion rate of prescribed intravenous perfusions. Speed of natremia correction was calculated using available natremia on the first day, the third day, and the last known natremia (*i.e.*, natremia at death, normalization, or loss to follow-up). Patients with no natremia improvement between entry and the last known natremia were defined as the no natremia improvement group. The plasma tonicity and osmolarity were calculated using the classical formulas: tonicity = ([Na] + [K]) ×2 + Glycemia; osmolarity = ([Na] + [K]) ×2 + Urea + Glycemia. Estimated glomerular filtration rate (eGFR) was calculated using the MDRD formula [17].

Hypotension requiring isotonic solute perfusion was defined as a mean blood pressure (MBP) <70 mmHg. Optimal correction rate of hypernatremia was defined as a decreasing rate between 0.5 to 1 mmol/L/h with a maximum of 12 mmol/L/day [3-6].

Statistical analysis was performed using SPSS version 1.0 and Stata 12. Continuous variables were expressed as means ± SD or as median with range (min, max), and categorical variables were reported as count and/or percentages. A Cox regression analysis was conducted to identify mortality risk factors. A p-value of <0.10 in the univariate analysis was chosen to define the variables to be entered into the selection procedure for the multivariable model. A step by step backward procedure was used to identify variables with a p-value <0.05 in the multivariate model. Natremia at admission was included in the multivariate model as an adjustment variable. Hazard ratios were expressed with 95% confidence intervals. All the tests were two-sided. Data with more than four groups were clustered to define correct Hazard Ratios. Research was conducted according to standard recommendations of the local ethics committee (Comité de Protection des Personnes Sud-Méditerranée II - http://www.cpp-sudmed2.fr) and followed the standards of the Helsinki Declaration. As defined by the French Public Health Code (Articles L1121-1 and R1121-2), no institutional review board and no written informed consent is required for "research in which all practices and products are used in the usual way, without any additional or unusual procedure for diagnosis or monitoring", thus, for retrospective studies.

## Results

Between January 2010 and January 2011, 54 753 admissions were recorded in our ED, 16 351 (29.8%) of them had a serum sodium dosing. Hypernatremia >145 mmol/L was found in 226 admissions, *i.e.*, 0.4% of admissions and 1.4% of admissions with natremia dosing.

Within the 226 hypernatremic admissions, 141 were between 146 and 149 mmol/L and not included in this study. The 85 admissions (82 patients) with severe hypernatremia, *i.e.*, ≥150 mmol/L (0.2% of admissions and 0.5% of admissions with natremia dosing), were included in this study.

**Figure 1:**
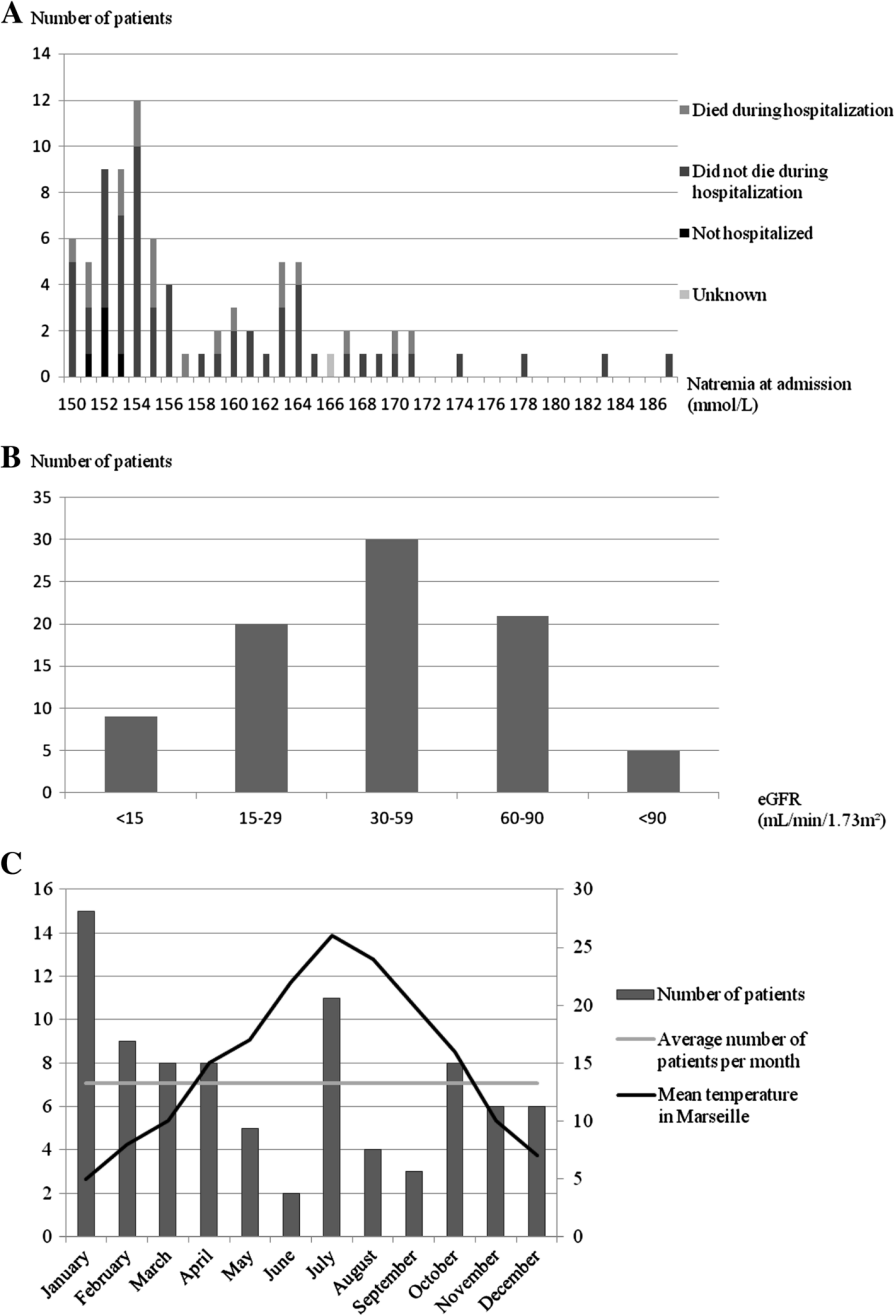
**Clinical and biological features of hypernatremic patients. A**. Natremia at admission and outcomes of hypernatremic patients. **B**. Renal function at admission. Renal function at admission was measured using estimated glomerular filtration rate (eGFR) estimated by Modification of Diet in Renal Disease formula (23). **C**. Month of occurrence of hypernatremia.

The main characteristics of these 85 admissions are reported in Table 1. At admission, mean natremia was 158 ± 8 mmol/L. For 68% of patients natremia was between 150 and 160 mmol/L and for 32% between 161 and 187 mmol/L (Figure 1A). Mean calculated plasmatic tonicity was 347 ± 25 mOsm/L, and mean calculated plasmatic osmolarity was 356 ± 26 mOsm/L. A urinary ionogram was performed for only 3 patients.

**Table 1:** Characteristics of hypernatremic patients and risk factors for death occurrence during hospitalization

**Characteristics**	**Total population (% or mean ± SD)**	**Death during hospitalization (% or mean ± SD)**	**No death during hospitalization (% or mean ± SD)**
	**n = 85**	**n = 19**	**n = 59**
**Age (years)**	79.7 ± 14	85.3 ± 10.7	77.2 ± 14.7
**Male**	44%	63%	41%
**Cause of admission**			
Asthenia	40%	53%	32%
Short breath	16%	32%	6%
Fever	12%	5%	15%
Diarrhea, vomiting or abdominal pain	9%	0%	14%
Neurologic symptom	8%	0%	12%
Trauma	4%	0%	3%
Hypotension	4%	5%	3%
Gastrointestinal bleeding	2%	5%	2%
Other			
**Place of origin and personal environment**			
Home	45%	42%	51%
Institution	50%	47%	46%
Home medical care	5%	11%	3%
**Season of occurrence**			
Winter	39%	47%	34%
Spring	20%	16%	24%
Summer	20%	26%	20%
Autumn	21%	11%	22%
**Dementia**	28%	31%	27%
**Treatment modifying renal water excretion**	23%	29%	24%
Loop diuretic	14%	22%	14%
Thiazide	4%	7%	4%
Potassium-sparing diuretic	3%	0%	4%
Lithium	1%	0%	2%
**Characteristics at admission**			
Systolic blood pressure (mmHg)	116 ± 29	94 ± 24	122 ± 26
Diastolic blood pressure (mmHg)	68 ± 18	55 ± 15	73 ± 17
Mean blood pressure (mmHg)	84 ± 20	67.8 ± 15.0	89.6 ± 17.8
Neurologic symptoms	66%	88%	59%
Extracellular dehydration	72%	88%	67%
Natremia (mmol/L)	158 ± 8	158.2 ± 6.5	158.5 ± 8.3
Calculated plasmatic tonicity* (mOsm/L)	347 ± 25	357 ± 28	346 ± 24
Calculated plasmatic osmolarity** (mOsm/L)	356 ± 26	365 ± 29	355 ± 25
Creatinine (μmol/L)	170 ± 108	225 ± 117	152 ± 95
eGFR*** (mL/min/1.73 m^2^)	45 ± 26	33.6 ± 24.2	48.9 ± 26.4
**Main diagnosis in emergency department**			
Infection	41%	39%	47%
Extracellular dehydration	33%	4%	16%
Gastrointestinal disease	6%	7%	5%
Shock (septic n = 3, hypovolemic n = 1)	5%	40%	16%
Acute pulmonary edema	2%	2%	0%
Other	13%	9%	16%
**Perfused solute**			
Hypotonic solute	64%	53%	65%
Isotonic solute	28%	42%	23%
No perfusion	1%	0%	2%
Unknown	7%	5%	11%
**Natremia correction speed:**			
Mean speed between entry and last known natremia (mmol/L/h)	−0.18 ± 0.21	−0.1 ± 0.15	−0.2 ± 0.22
No natremia improvement	27%	44%	21%

Seven patients who were lost to follow-up on the day of admission were not included in statistical analysis. *Calculated plasmatic tonicity = ([Na] + [K]) ×2 + Glycemia. **Calculated plasmatic osmolarity = ([Na] + [K]) ×2 + Urea + Glycemia. ***Glomerular filtration rate (eGFR) was estimated with the simplified Modification of Diet in Renal Diseases formula [23].

Mean creatinine at admission was 170 ± 108 μmol/L, but no information was available with regard to acute or chronic renal failure. Using the MDRD formula, which is appropriate for chronic renal failure, mean eGFR was 45 ± 26 mL/min/1.73 m^2^; 94% of patients had an eGFR <90 mL/min/1.73 m^2^, 69% an eGFR <60 mL/min/1.73 m^2^, and 34% an eGFR <30 mL/min/1.73 m^2^ (Figure 1B).

For 47% of patients, main diagnosis recorded at the ED was a medical disease frequently associated with water loss: infection (pulmonary n = 20, urinary n = 11, other n = 4), gastrointestinal disease (diverticulitis n = 1, diarrhea n = 2, gastroenteritis n = 2) (Table 1).

Surprisingly, hypernatremia was more frequent in winter (39% of cases) than in spring (20%), autumn (21%), and notably summer (20%). In summer, a short peak was observed in July, the hottest month of 2010 in Marseille (Figure 1C).

Rehydration treatment prescribed at ED is reported in Table 2. Intravenous perfusions was inappropriate for 45% of patients with MBP <70 mmHg who received a hypotonic solute, and for 22% with MBP ≥70 mmHg who received isotonic solute or were not perfused. In 74% of the medical files perfusion speed was missing; in 6% the perfusion setting by the nurse was not reported.

**Table 2:** Initial fluid replacement strategy on arrival at emergency department

**Treatments**	**n = 85**
**Perfused solute**	
Hypotonic solute	64%
Isotonic solute	28%
0.9% sodium chloride	67%
Ringer’s lactate*	21%
Hydroxyethyl starch 130/0.4**	12%
No perfusion	1%
Unknown	7%
**Perfused solute according to mean blood pressure**	
MBP <70 mmHg	24%
Hypotonic solute	45%
Isotonic solute	55%
MBP ≥ 70 mmHg	69%
Hypotonic solute	71%
Isotonic solute	20%
No perfusion	2%
Unknown	7%
Unknown MBP	7%
**Other**	
No prescribed perfusion rate	74%
Perfusion not reported in file by the nurse	6%

*Ringer’s lactate contains sodium lactate 3.2 g/L and sodium chloride 6 g/L.

**Hydroxyethyl starch 130/0.4 contains hydroxyethyl starch 130 60 g/L and sodium chloride 9 g/L.

As described in the Methods, hypotension requiring isotonic solute perfusion was defined as a mean blood pressure (MBP) <70 mmHg. Thus, intravenous perfusion was inappropriate for almost half of patients with MBP <70 mmHG who received hypotonic solutes, and for nearly a quarter when MBP ≥70 mmHg who received isotonic solutes or were not perfused.

After their ED evaluation, 78 of the 85 patients (92%) were hospitalized (Figure 1A, Table 3), 5 returned to their previous institution, 1 died in the ED, and 1 patient's outcome is unknown. Mean hospitalization duration was 13.1 ± 13 days.

**Table 3:** Outcome and natremia evolution in the emergency department and during hospitalization

**Outcome**	**n = 85**
**All patients**	
**Outcome after emergency department**	
Hospitalization	92%
Back to institution	6%
Died at emergency department	1%
Unknown	1%
**In hospital mortality**	24%
**Mean time to death** (days)	5.8 ± 6
**Hospitalized patients**	**n = 78**
**Natremia at admission** (mmol/L)	158 ± 8
**On the next day** (n = 59)	
Time lag between initial natremia and next day natremia	17 h21 ± 6 h28
Mean natremia (mmol/L)	156 ± 7
Mean correction speed (mmol/L/h)	−0.18 ± 0.39
Worsened natremia	34%
Same natremia as at admission	8%
Improved natremia	58%
At a less than 0.5 mmol/L/h speed	65%
At a 0.5 to 1 mmol/L/h speed	32%
At a more than 1 mmol/L/h speed	3%
**On the third day** (n = 53)	
Time lag between initial natremia and 3^rd^ day natremia (days)	2,47 ± 0.72
Mean natremia (mmol/L)	150 ± 8
Mean correction speed (mmol/L/h)	−0.15 ± 0.19
Worsened natremia	11%
Same natremia as at admission	6%
Improved but not normalized natremia	51%
Normalized natremia	32%
**Mean hospitalization time** (days)	13.1 ± 13

Data are given as mean ± standard deviation or percentages.

Natremia evolutions and patient outcomes are reported in Table 3. Compared to admission, natremia on the next day was worse in 34%, identical in 8%, and improved in 58% of patients. For 65% of patients with improvement, correction was <0.5 mmol/L/h, for 32% between 0.5 and 1 mmol/L/h, and >1 mmol/L/h for 1 patient who did not die during hospitalization. Overall, only 19% of patients improved their natremia with the recommended rate of 0.5 to 1 mmol/L/h.

During hospitalization, 57% of patients normalized their natremia. Their mean rate of lowering was −0.24 ± 0.2 mmol/L/h in 4.87 ± 3.28 days (from 11 hours to 14 days). Among them, 39% had normal natremia on the third day and 80% on the 7^th^ day. When leaving the hospital, 27% of patients had no natremia improvement and 19% had not normalized their natremia.

Hospital mortality rate was 24% (19 patients), occurring 5.8 ± 7 days after their arrival in ED, 47% patients died on the first two days (including the patient who died in ED); 68% in the first week; and 32% after 7 days of hospitalization. Last natremia before death was 155 ± 9 mmol/L, with a lowering speed of −0.1 ± 0.15 mmol/L/h. Only 11% of patients had natremia normalized before death.

Because perfusion speed was unknown in 74% of the cohort, we were not able to analyze the link between perfusion speed and natremia correction speed. Taking this limit into account, the type of solute prescribed was not associated with the correction speed during the first day (hypotonic solute −0.2 ± 0.37 mmol/L/h *versus −*0.21 ± 0.44 mmol/L/h with isotonic solute, p = 0.98).

In monovariate analysis, risk factors at admission for in-hospital death were (Table 4) older age (p = 0.04), presence of neurologic symptoms (p = 0.03), lower systolic blood pressure (p < 0.001), lower diastolic blood pressure (p = 0.001), lower MBP (p < 0.001), higher creatinine (p = 0.004), lower eGFR (p = 0.03), infection as the main diagnosis in the ED (p = 0.004), and having no natremia improvement (p = 0.02).

**Table 4:** Factors associated with death during hospitalization: univariate and multivariate analysis using a Cox regression model (n = 78)

	**Monovariate analysis**			**Multivariate analysis**		
	**p**	**HR**	**CI 95%**	**p**	**Adjusted HR**	**CI 95%**
Age	0.04	1.05	1.00-1.10			
Male gender	0.06	2.47	0.97-6.28	0.007	5.65	1.60-19.90
Place of origin and personal environment:						
Home	Ref.	Ref.	Ref.			
Institution or home medical care	0.65	1.24	0.50-3.08			
Season of occurrence:						
Winter	Ref.	Ref.	Ref.			
Spring	0.36	0.54	0.15-2.00			
Summer	0.79	0.86	0.29-2.59			
Autumn	0.21	0.38	0.08-1.75			
Dementia	0.54	1.40	0.48-4.04			
Treatment modifying renal water excretion	0.95	1.04	0.32-3.33			
Characteristics at admission:						
Systolic blood pressure (mmHg) †	<0.001	0.97	0.95-0.98			
Diastolic blood pressure (mmHg) †	0.001	0.95	0.92-0.98			
Mean blood pressure	<0.001	0.95	0.92-0.97	<0.001	0.92	0.91-1.06
Neurologic symptoms	0.03	5.22	1.20-22.78			
Extracellular dehydration	0.07	4.02	0.92-17.63			
Natremia (mmol/L)	0.88	1.01	0.94-1.07	0.64	0.98	0.88-0.95
Calculated plasmatic tonicity (mOsm/L) †	0.06	1.02	1.00-1.04			
Calculated plasmatic osmolarity (mOsm/L)	0.09	1.02	1.00-1.03			
Creatinine (μmol/L)	0.004	1.01	1.00-1.01			
eGFR (mL/min/1.73 m^2^) †	0.03	0.98	0.95-1.00			
Main diagnosis in emergency department:						
Extracellular dehydration	Ref.	Ref.	Ref.			
Infection	0.04	4.21	1.09-16.29			
Other						
Perfused solute:	0.18	2.46	0.67-9.10			
Hypotonic solute	Ref.	Ref.	Ref.			
Isotonic solute, no perfusion or unknown	0.44	1.43	0.58-3.52			
Natremia correction speed:						
Mean speed between entry and last known natremia (mmol/L/h) †	0.10	18.79	0.59-597.13			
No natremia improvement	0.02	3.12	1.22-7.96	<0.001	10.29	3.12-33.96

Factors indicated by † were not included in the multivariate analysis because a linked factor was already included in the analysis.

In multivariate analysis, male gender (p = 0.007; HR = 5.65 95% CI [1.60-19.90]), lower MBP (p < 10^-3^; HR = 0.92 95% CI [0.88-0.95]), and slow mean correction speed to last known natremia (p < 10^-3^; HR = 10.29 95% CI [3.12-33.96]) were associated with death occurrence during hospitalization.

## Discussion

Our study describes initial presentation, management, and outcomes of severely hypernatremic patients (≥150 mmol/L) in the ED and during the following hospitalization. Relative to the 50 000 patients hospitalized each year in our ED, severe hypernatremia is a rare event. However, unlike in-hospital acquired hypernatremia, most patients with hypernatremia at hospital admission receive initial treatment in the ED. To our knowledge, very few studies have reported on presentation, management and outcome of severely hypernatremic patients upon their arrival to the hospital (Table 5) [1,2,18]. Arampatzis et al. reported hypernatremia >145 mmol/L in 0.9% of patients admitted toED and 1.5% of patients with natremia dosing, and severe hypernatremia ≥150 mmol/L in 0.1% of admissions and 0.2% of patients with natremia dosing, which is similar to our results.

**Table 5:** Studies reporting out-of-hospital acquired hypernatremia

	**Liamis et al. **[15]	**Arampatzis et al. **[1]	**Arampatzis et al. **[2]	**Baralla et al.**
Publication year	2008	2012	2012	2013
Country	Greece	Switzerland	Switzerland	France
Department	Internal medicine	Emergency	Emergency	Emergency
Hypernatremia definition (mmol/L)	> 148	> 142	≥ 150	≥ 150
N=	55	400	74	85
Mean age (years)	76.3 ± 12.2	53 ± 22	NA	79.7 ± 14
Males	40%	59%	62%	44%
Mean Na (mmol/L)	160.4 ± 9.9	144 ± 2	152	158 ± 8
Mortality during hospitalization	28%	NA	28%	24%

Arampatzis et al. published two studies in the same year. Patients of the second study with hypernatremia defined as ≥150 mmol/L were a subgroup of the former larger study, with an unclassical hypernatremia definition.

In our study, hypernatremic patients were old (mean age 79.7 years-old), lived in medical institutions or at home with medical care (55%), and had cognitive impairment (28% had dementia), which are situations of thirst impairment and/or limited access to water and at high risk for dehydration [19-21]. Moreover, for 47% of patients with severe hypernatremia ≥150 mmol/l, the main diagnosis was a situation associated with water loss, and 23% of patients had a treatment modifying renal water excretion.

Hot weather is an understandable risk factor for hypernatremia, and summer heat is regularly evoked [20]. Surprisingly, in our study, hypernatremia was more frequent in winter. Several hypotheses can be proposed: less important care given to patient’s hydration in winter than in summer [22], a higher infection rate in winter [23]. A peak of hypernatremia was also noted in July, the hottest month in our city in 2010 and the 6^th^ hottest July since 1900 with a temperature 1.9°C above normal (Source Météo France, http://climat.meteofrance.com). Clearly, heat is an important risk factor for hypernatremia, but our study also highlights that an adequate water intake in the elderly is important all year.

Neurological symptoms are frequent in hypernatremia, and we report that 66% of patients had at least one neurological symptom at admission. This is higher than the 38% reported by Arampatzis et al., who do not specify how many patients had previous dementia [2]. Extracellular dehydration was present in most patients (72%) and could be responsible for the hypotension and the renal impairment observed in 69% of patients (eGFR below 60 mL/min/1.73 m^2^).

This is the first study analyzing natremia correction speed and treatment given to hypernatremic patients in ED. We show that under-correction is frequent in the ED and in the first few days following admission: on the day after admission, 42% had a worsened or identical natremia; and on the third day following admission, natremia was normalized in only 32% of patients. More than one patient out of four had no natremia improvement between entry and last follow-up. Overcorrection is rare and only one patient with lithium induced nephrogenic insipid diabetes had a correction speed >1 mmol/L/h. He had a good evolution and returned to his institution after hospitalization. Optimal speed of natremia correction is not clearly established but experts agree that the correction speed should not exceed 1 mmol/L/h, without a given lower limit [3-6]. Nevertheless, it is important not to prolong hypernatremia duration which induces cerebral suffering and the risk of neurological sequels, decreases cardiac contractility, increases peripheral insulin resistance and impairs hepatic neoglucogenesis [24]. We could not determine whether insufficient correction of hypernatremia was due to a low speed of perfusion because perfusion speed was missing in 74% of the cohort. This high percentage of missing data might reflect the lack of precision of medical prescriptions or the lack of transcription of the nurse on the medical file. Moreover, this speed must be adjusted to total body water deficit extrapolated from natremia and body weight, but weight was recorded for only 3 patients. Also, water loss was evaluated from urinary ionogram in only 3 patients. Besides the too slow perfusion speed, mistakes in the type of solute chosen for rehydration could at least partly explain under-correction, as 22% of patients with MBP ≥70 mmHg either received isotonic solute or were not perfused.

Despite the non classical management of hypernatremia, the mortality rate of our cohort (24%) is similar to those previously reported, suggesting that our practices are comparable to those of other EDs (Table 5). In intensive care units, hypernatremia occurrence is a known mortality risk factor but patients are different and hypernatremia is frequently iatrogenic [24]. Indeed, the consequences of cerebral edema differ with age, with a major risk at younger age when the size ratio of brain to cranial volume is high [25]. In our study, hypernatremic patients were old and often had dementia, two conditions associated with cerebral atrophy. Moreover, we show that hypovolemia signs (extracellular dehydration, hypotension, and renal insufficiency) are the principal risk factors for death during hospitalization, suggesting that associated illness and medical status are the major risk factors for death in patients with hypernatremia acquired outside the hospital. This hypothesis is supported by the short delay before death (68% in the first week, most of them during the first 2 days). Three of the 4 patients with a diagnosis of shock at ED died during hospitalization.

In this study, we did not find a statistical link between the level of hypernatremia and mortality. Higher calculated plasmatic tonicity and calculated plasmatic osmolarity were associated with death in the monovariate model (Table 4), but not in the multivariate analysis. The 85 cases analyzed represent a relatively small cohort and there were numerous confounding factors such as loss to follow-up, associated comorbidities, and associated acute illness. The small cohort size also probably explains why the relationship between hypernatremia and death did not reach statistical significance. Moreover, our monocentric study might reflect local practices and a local effect. Nevertheless, in our study, a too slow natremia correction speed was associated with death. This is the first study reporting that under-correction of hypernatremia is associated with an increased mortality. Time spent with intracellular dehydration could be more important than the severity of hypernatremia or hyperosmolarity. In hyponatremia, it is well known that a too high correction speed can lead to osmotic demyelination. In hypernatremia, similar recommendations are given, but physicians should also be aware that a too slow correction rate could increase the risk of death.

## Conclusion

This is the first study assessing outcome of hypernatremic patients in an ED according to the provided treatment. It suggests that medical management of hypernatremic patients must be improved regarding evaluation (weight, diuresis, urinary ionogram) and management (type of solute, perfusion speed) and that a too slow correction speed is associated with an increased risk of death regardless of initial natremia.

## Competing interests

The authors declare that they have no competing interests.

## Authors’ contributions

SB and CBa collected and analyzed the data and wrote the paper. DT had the idea and supervised the study. CBu provided the biological data. YB, MA and PM gave advice to improve the study. AL did the statistical analysis. HVC supervised the study and guided SB and CBa in the paper writing. Each author read and corrected the paper. All authors read and approved the final manuscript.

## Pre-publication history

The pre-publication history for this paper can be accessed here:

http://www.biomedcentral.com/1471-2369/15/37/prepub
